# Plasma metabolite association profiles for type 2 diabetes genetic clusters in Finnish men

**DOI:** 10.1007/s00125-026-06710-9

**Published:** 2026-03-18

**Authors:** Ruyi Peng, Lei Liu, Xiaomeng Chu, Zhijie Xia, Qi Fu, Lilian Fernandes Silva, Xiaolong Ji, Xinxian Hu, Yuxi Liang, Jack Li, Brady Ryan, Debraj Bose, Heather M. Stringham, Jean Morrison, Xiaoquan Wen, Laura J. Scott, Charles F. Burant, Eric Fauman, Tao Yang, Michael Boehnke, Markku Laakso, Xianyong Yin

**Affiliations:** 1 Department of Epidemiology, School of Public Health, Lianyungang Medical-Education Innovation and Research Center, Nanjing Medical University, Nanjing, China; 2 Jiangsu Key Laboratory of Molecular Targets and Intervention for Metabolic Diseases, Nanjing, China; 3 Department of Endocrinology and Metabolism, The First Affiliated Hospital of Nanjing Medical University, Nanjing, China; 4 Institute of Clinical Medicine, Internal Medicine, University of Eastern Finland, Kuopio, Finland; 5 Department of Biostatistics and Center for Statistical Genetics, University of Michigan, Ann Arbor, MI USA; 6 Department of Internal Medicine, University of Michigan, Ann Arbor, MI USA; 7 Internal Medicine Research Unit, Pfizer Worldwide Research, Development and Medical, Cambridge, MA USA

**Keywords:** Genetic cluster, Metabolomics, Polygenic risk score, Type 2 diabetes

## Abstract

**Aims/hypothesis:**

A recent study has suggested eight clusters of genetic variants associated with type 2 diabetes. We aimed to characterise metabolite associations for these eight clusters.

**Methods:**

We constructed type 2 diabetes overall and cluster-partitioned polygenic risk scores (PRSs) in 10,015 Finnish men with 979 named plasma metabolites measured in Metabolon HD4 mass spectrometry platform. We evaluated metabolite–PRS associations using linear regression. We also performed a mediation analysis to examine whether metabolites statistically accounted for part of the association between genetic risk and incident type 2 diabetes that developed in a mean of 13.6 years’ follow-up.

**Results:**

We identified 337 metabolites significantly associated with type 2 diabetes genetic risk, including 242 exclusive to cluster-partitioned PRSs. Of the significant metabolites, 26 exhibited significantly heterogeneous associations across clusters. We identified significant enrichment for 33 metabolic pathways among the cluster-associated metabolites. Notably, metabolites for the two pancreatic beta cell-related clusters exhibited enrichment in distinct pathways: the beta cell + proinsulin (PI) cluster in fructose, mannose and galactose metabolism; and the beta cell − PI cluster in branched-chain amino acid metabolism. Mediation analysis suggested that >50% of the associated metabolites showed patterns statistically consistent with a mediating role in the associations between PRSs and incident type 2 diabetes.

**Conclusions/interpretation:**

This study underscores the value of type 2 diabetes clustering and highlights metabolic heterogeneity across clusters. The findings have the potential to guide personalised interventions.

**Data availability:**

The datasets generated during and/or analysed in the current study are available in dbGaP (accession ID: phs000743.v4.p1 and phs004033.v1.p1).

**Graphical Abstract:**

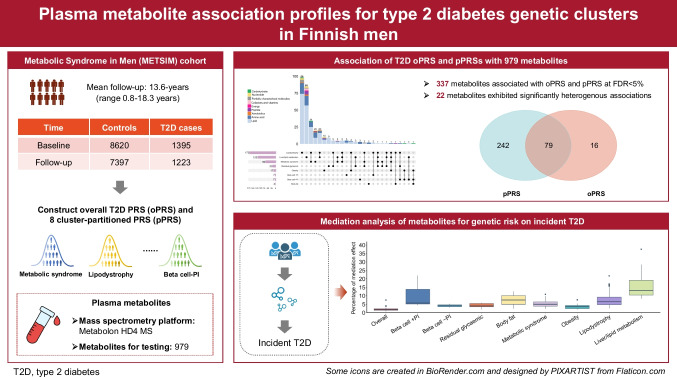

**Supplementary Information:**

The online version of this article (10.1007/s00125-026-06710-9) contains peer-reviewed but unedited supplementary material.


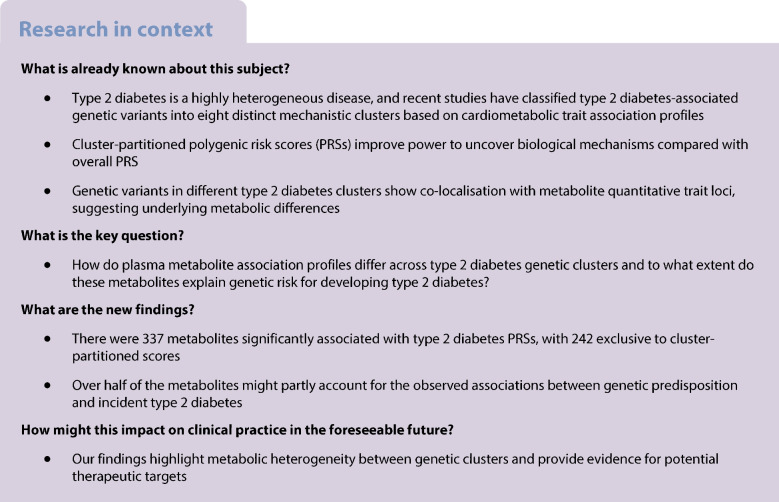


## Introduction

Type 2 diabetes is a common metabolic disease. It exhibits substantial heterogeneity in genetic susceptibility, disease pathophysiology, clinical manifestation and treatment response, posing challenges for personalised treatment and suggesting the need for precise disease clustering [1, 2].

Recently, Suzuki et al performed the largest type 2 diabetes genome-wide association study (GWAS) meta-analysis to date, identifying associations with 1289 genetic variants [3]. Through the association profiles of these 1289 genetic variants with 37 cardiometabolic traits, they identified eight non-overlapping mechanistic clusters of type 2 diabetes genetic variants: beta cell + proinsulin (PI); beta cell – PI; residual glycaemic; body fat; the metabolic syndrome; obesity; lipodystrophy; and liver and lipid metabolism clusters. These eight genetic clusters demonstrated differential enrichment for cell-type-specific open chromatin regions and associations with macrovascular disease outcomes [3]. Genetic variants within the eight clusters showed co-localisations with distinct metabolite genetic regulation, suggesting metabolic pathways underlying genetic variants within clusters [4]. However, this study detected significant co-localisations for only 22.0% of genetic variants within the eight type 2 diabetes clusters [4]. Metabolic pathways remained unexplored for most of the genetic variants within the eight clusters.

A polygenic risk score (PRS), which aggregates effects across genetic variants, can improve power for uncovering disease mechanisms when linking with molecular data. In addition to overall PRS (oPRS) which treats type 2 diabetes as a genetically homogeneous disease and aggregates effects across all genetic variants, genetic cluster-partitioned PRSs (pPRSs) have also been deployed to capture type 2 diabetes biological processes [5–7]. Compared with oPRS, pPRSs demonstrated extra benefit for uncovering type 2 diabetes biomarkers and biological pathways [8]. No study has yet systematically investigated metabolite association profiles for the pPRSs of the eight type 2 diabetes genetic clusters. We aimed to systematically evaluate associations between plasma metabolites and genetic risk of the eight type 2 diabetes clusters in the Metabolic Syndrome in Men (METSIM) study, and to quantify the extent to which these metabolites may partly account for the associations.

## Methods

### METSIM study

**Fig. 1:**
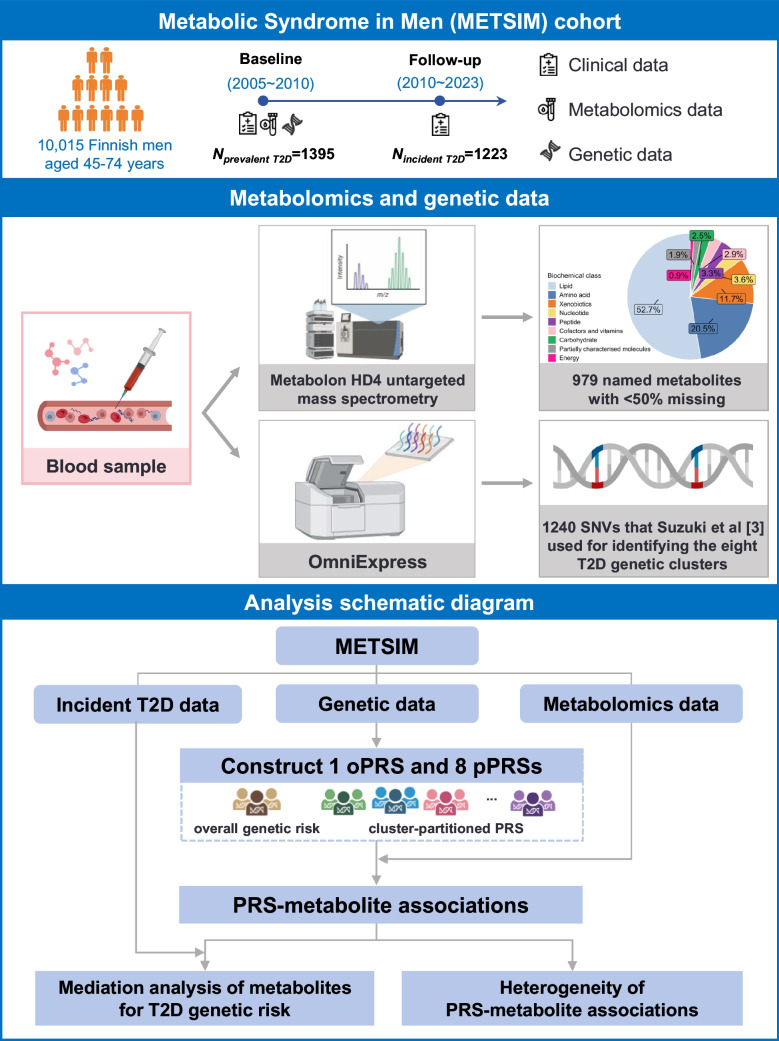
Study design overview. All icons in this plot are created in BioRender.com or designed by Freepik from Flaticon.com. SNV, single nucleotide variant; T2D, type 2 diabetes

METSIM is a longitudinal cohort designed to investigate risk factors for cardiometabolic diseases in Finns [9]. It randomly recruited 10,197 Finnish men aged 45–74 years at baseline from Kuopio between 2005 and 2010. Given the higher prevalence of cardiovascular diseases in men, METSIM focused exclusively on men (with self-reported gender). Participants were followed up through clinical revisits and regular linkage to Finnish national registries [9], with the most recent contact being in June 2023 (Fig. 1 and electronic supplementary material [ESM] Table 1). At baseline, we collected biological samples and deep phenotypes from all participants (e.g. BMI, medication prescriptions, alcohol drinking and OGTT). For nearly all participants, we ascertained genome-wide genotype data using the Human OmniExpress-12v1_C BeadChip (OmniExpress) [9] and performed genotype imputation using the METSIM integrative reference panel [10]. This study was approved by the Ethics Committee at the University of Eastern Finland. All participants provided written informed consent.

### METSIM metabolomics data

We performed untargeted metabolic profiling in the baseline plasma samples for almost all METSIM participants using mass spectrometry technology (Metabolon, Durham, NC) [10]. We identified 1540 metabolites including 281 with unknown chemical identities. We excluded individuals with missing information on BMI, smoking or lipid-lowering medication use status, and those with type 1 diabetes at baseline. In METSIM, type 2 diabetes was diagnosed using laboratory tests or electronic medical records. We performed quality control on the genotype and metabolomic data as described [10]. We excluded the 281 unnamed and 280 named metabolites with a missing rate greater than 50%. The remaining 979 named metabolites belonged to eight biochemical classes and 105 metabolic pathways (ESM Table 2). For each metabolite, we imputed missing values using the *k*-nearest neighbours algorithm (KNN, *k*=10) [10].

### PRS construction

To quantify type 2 diabetes genetic risk, we calculated an oPRS and eight pPRSs for METSIM participants based on the genetic associations and the eight genetic clusters identified by Suzuki et al [3]. We calculated PRS as:

$${\mathrm{PRS}}_{\mathrm{k}}=\sum_{\mathrm{j}=1}^{\mathrm{M}}{\mathrm{a}}_{\mathrm{kj}}{\mathrm{W}}_{\mathrm{j}}$$

where W_j_ is the natural logarithm of the reported association OR for genetic variant j; a_kj_ is the imputation dosage of the risk allele for variant j in participant k; and M is the total number of overall genetic variants or the variants within the respective genetic cluster.

Of the 1289 variants identified by Suzuki et al [3], 1240 were available in METSIM imputed genotypes (ESM Table 3); 46 were monomorphic or extremely rare (minor allele frequency <0.005%) in Finns [11] and three were imputed with low confidence in METSIM (ESM Table 4).

### Identification of type 2 diabetes PRS–metabolite associations

To identify metabolites associated with type 2 diabetes genetic risk, we tested metabolite associations with both type 2 diabetes oPRS and pPRSs for the eight clusters in METSIM participants using linear regression. For PRS, we performed *z* score standardisation, regressed out the first ten genetic principal components (PCs) and standardised the residuals using *z* score standardisation [12]. For each of the 979 metabolites, we regressed out covariates: Metabolon experiment batch; study age; BMI; smoking status; alcohol consumption; lipid-lowering medication usage; the first ten genetic PCs; and type 2 diabetes status at baseline. This was followed by inverse normalisation [12]. We identified significant PRS–metabolite associations at false discovery rate (FDR) <5%. To evaluate the robustness of these associations, we repeated the PRS–metabolite association analyses in participants without baseline type 2 diabetes (*n*=8620), participants with normal glucose tolerance (NGT; *n*=5744) and participants with baseline type 2 diabetes (*n*=1395).

For PRS-associated metabolites, we tested for associations with prevalent and incident type 2 diabetes in METSIM after adjusting for study age, BMI and Metabolon batch using logistic regression and Cox proportional hazards models, respectively.

For lipids, we extracted their total acyl chain carbon number and total number of double bonds, followed by calculating Spearman’s rank correlation between these chemical features and their association coefficients with PRS. We restricted this analysis to metabolic pathways including five or more lipids.

### Enrichment analysis of biochemical class and metabolic pathways

To evaluate the enrichment or depletion of PRS-associated metabolites across biochemical classes and metabolic pathways, we compared the proportions of metabolites belonging to each class or pathway in PRS-associated metabolites within all the 979 tested metabolites. We applied a χ^2^ goodness-of-fit test to assess whether the observed distribution significantly deviated from expectation (FDR<5%). We calculated an enrichment fold as the ratio of proportion of PRS-associated metabolites in a given class or pathway and proportion of all the 979 metabolites in that class or pathway.

### Mediation analysis

To examine whether metabolites may partly account for the association between polygenic risk and type 2 diabetes, we conducted a statistical mediation analysis for the significant PRS–metabolite associations in the most recent follow-up data (June 2023) for METSIM participants without baseline type 2 diabetes (Fig. 1 and ESM Table 1). We used PRS as the exposure, metabolites as the putative mediators and incident type 2 diabetes as the outcome. This analytical approach rests on the assumptions of a correctly specified causal ordering (PRS–metabolite–incident type 2 diabetes) and the absence of unmeasured confounding of the metabolite–incident type 2 diabetes relationship. We adjusted for the same covariates except baseline type 2 diabetes status and transformed metabolomics data and PRSs as described under Identification of type 2 diabetes PRS–metabolite associations, above. To facilitate interpretation, we excluded PRS–metabolite–type 2 diabetes triplets exhibiting a suppression effect, where the indirect and direct effects of PRS on incident type 2 diabetes act in opposite directions, from downstream analysis [13].

To evaluate the robustness of the statistical mediation effects for pPRS, we reran the mediation analysis for pPRS–metabolite associations after adjusting for the type 2 diabetes oPRS or baseline HbA_1c_ or fasting glucose levels. For each metabolite with an indirect effect estimate consistent with potential mediation, we calculated the percentage statistically accounted for in the type 2 diabetes incident risk association as: (indirect effect / total effect) × 100%. We performed mediation analysis using the R package mediation (version 4.5.0) [14]. We identified metabolites with statistically significant indirect effects at FDR<5%.

To evaluate the potential joint mediation effects of multiple metabolites, we randomly divided METSIM participants without baseline type 2 diabetes into a discovery set (70%) and a validation set (30%). In the discovery set, to account for phenotypic correlations among metabolites, we applied elastic-net regression to select independently-associated metabolites for each pPRS, followed by calculating a metabolite score for each participant in the validation set using the derived association weights. We subsequently performed a mediation analysis to evaluate the potential role of the metabolite score in the association of pPRS with incident type 2 diabetes.

### Drug target analysis

We queried DrugBank to assess whether putative causal genes nominated for metabolites that showed a pattern statistically consistent with a mediating role are known drug targets [15]. To evaluate whether the observed overlap between the putative causal genes and drug-related genes expected by chance, we performed a gene set enrichment analysis using the over-representation analysis method against the DrugBank drug–gene interaction database in WebGestalt 2019 (http://www.webgestalt.org/, accessed 26/11/2025) [16, 17]. All analyses used the human genome as the reference set with default parameters. We identified significant enrichment at FDR<5%.

## Results

### Study design

After quality control, we identified 10,015 participants with both genotype and metabolomics data available in METSIM. Of these participants, 1395 had prevalent type 2 diabetes at baseline and 1223 had incident type 2 diabetes during a mean of 13.6-years follow-up (range 0.8–18.3 years). We implemented a three-stage design in this study (Fig. 1). First, we tested for associations between type 2 diabetes oPRS and eight pPRSs and the 979 named plasma metabolites. We then characterised heterogeneity of metabolite associations across the eight type 2 diabetes genetic clusters. Finally, for the associated metabolites, we evaluated whether their association patterns were statistically consistent with a mediating role between genetic risk and incident type 2 diabetes developed in 1223 participants during follow-up.

### Metabolite associations with type 2 diabetes oPRS

**Fig. 2:**
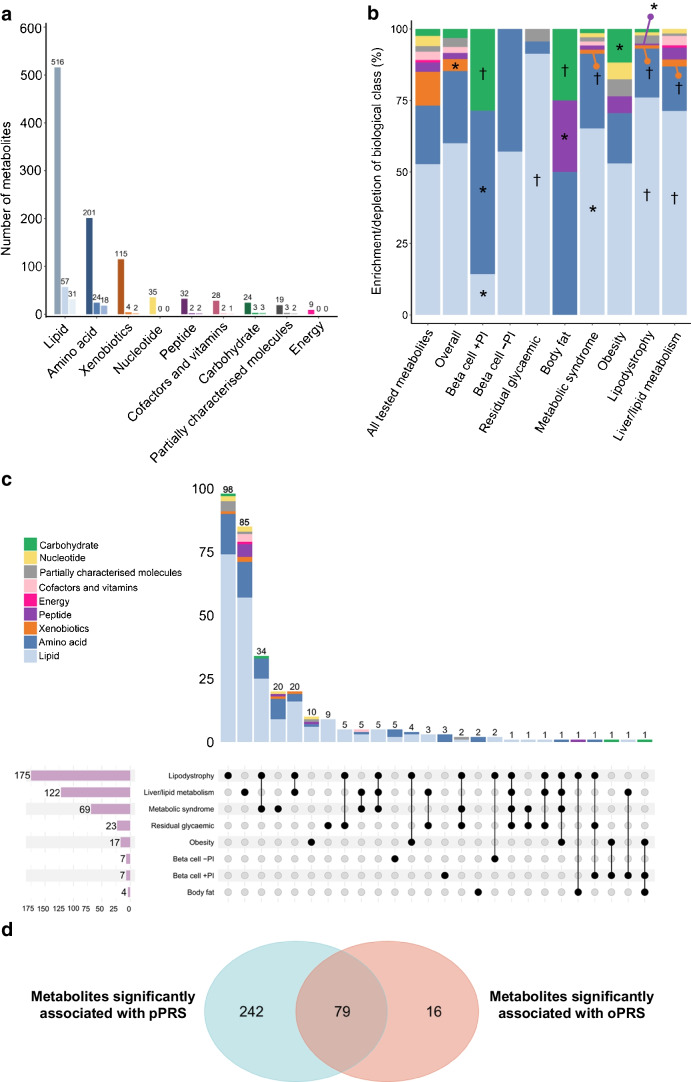
Metabolite associations with type 2 diabetes overall and cluster-partitioned genetic risk. (**a**) Number of metabolites associated with type 2 diabetes oPRS. The *x*-axis shows different biochemical classes of metabolites and the *y*-axis shows the number of metabolites. Colours from dark to light represent all test metabolites, metabolites with significant associations and metabolites with significant mediation effects, respectively. (**b**) Enrichment of biochemical class in PRS-associated metabolites. *p<0.05, ^†^corrected significant FDR<0.05, enrichment or depletion in the respective class. (**c**) Upset plot of associated metabolites across the eight type 2 diabetes genetic clusters. The total number of metabolites associated with each cluster is shown, along with the number of associated metabolites shared between clusters and the intersections of specific clusters. (**d**) Venn diagram showing the number of metabolites commonly and distinctly associated with type 2 diabetes pPRS and oPRS

We identified 95 significant associations of metabolites with type 2 diabetes oPRS at FDR<5% (Fig. 2a and ESM Table 2). Of the 95 significant metabolite associations, 81 (85.3%) showed consistent effect directions in METSIM participants with type 2 diabetes at baseline (ESM Fig. 1).

The 95 associations arose among metabolites in seven biochemical classes (Fig. 2a). Compared with the overall set of 979 metabolites, the 95 metabolites associated with the type 2 diabetes oPRS were nominally depleted in xenobiotics (*p*=0.023) and significantly enriched in six metabolic pathways (FDR<5%; Fig. 2b and ESM Table 5): monounsaturated acyl carnitine metabolism; acyl choline metabolism; lactoyl amino acids; lysophospholipids; lysoplasmalogens; and plasmalogens. These metabolic pathway findings were consistent with previous reports [18–22] and reinforce their potential roles in type 2 diabetes pathogenesis.

### Metabolite associations with type 2 diabetes pPRS

We identified 321 metabolites significantly associated with at least one pPRS at FDR<5%, resulting in 424 pPRS–metabolite association pairs (Fig. 2c and ESM Table 2). Of these 424 pPRS–metabolite associations, 387 (91.3%) achieved consistent effect directions in METSIM participants with type 2 diabetes at baseline (ESM Fig. 2). For the eight pPRSs, we identified 4–175 significantly associated metabolites (median = 20, mean = 53), with the largest and smallest number of metabolites for the lipodystrophy (*n*=175) and the body fat (*n*=4) clusters, respectively (Fig. 2c). We noted a pattern of negative correlation between the number of associated metabolites and the number of type 2 diabetes genetic variants within each cluster (Pearson *r*=−0.58, *p*=0.13; ESM Fig. 3). Compared with the other clusters, the liver and lipid metabolism cluster and the lipodystrophy cluster contained fewer genetic variants but achieved larger numbers of significant metabolite associations, suggesting a regulatory role of genetic variants within these two type 2 diabetes clusters on a wide spectrum of plasma metabolites.

In total, we identified 337 metabolites significantly associated with either the oPRS or the eight pPRSs (Fig. 2d). Of them, 227 (67.4%) were significantly associated with either prevalent or incident type 2 diabetes (ESM Table 6). We identified significant correlations of lipids’ numbers of total acyl chain carbon and of double bonds with their association coefficients with oPRS and pPRSs (ESM Fig. 4). For example, we identified an inverse relationship between the association coefficients of diacylglycerols with the pPRS for the lipodystrophy cluster and both their total acyl chain carbon and double bond numbers (ESM Fig. 4), concordant with a previous report [23].

### Extra insights gained through testing metabolite associations with type 2 diabetes pPRSs

Of the 321 metabolites significantly associated with pPRSs, 79 also showed significant associations with type 2 diabetes oPRS (Fig. 2d). The remaining 242 metabolites only achieved significant association with pPRSs and 175 of these metabolites failed to show even nominal significance with oPRS (*p*≥0.05), suggesting that stratifying genetic risk could reveal additional type 2 diabetes metabolic characterisation. To assess the additional information afforded by the pPRSs over the oPRS, we reran the associations of type 2 diabetes pPRS with all the 979 metabolites with adjustment for the oPRS. These pPRS–metabolite associations were strongly consistent (Pearson *r*=0.97; ESM Fig. 5). Of the 424 pPRS–metabolite pairs, 301 (71.0%) remained statistically significant even after adjusting for oPRS (FDR<5%), indicating that these pPRS–metabolite associations are not fully explained by the oPRS (ESM Table 2).

### Heterogeneity in pPRS–metabolite associations across type 2 diabetes genetic clusters

**Fig. 3:**
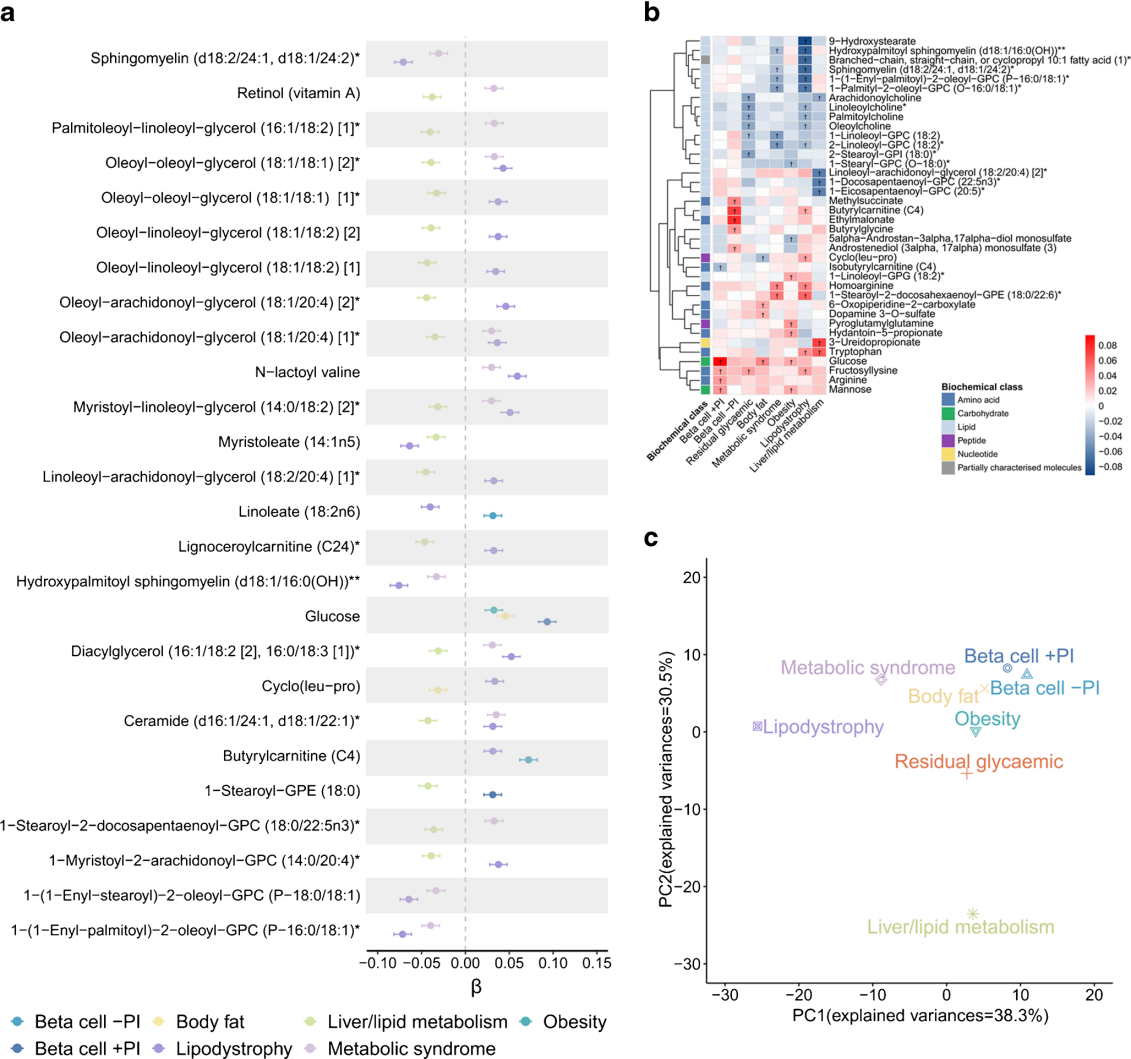
Heterogeneity of metabolite associations across type 2 diabetes genetic clusters. (**a**) Metabolite associations with significant heterogeneity across type 2 diabetes genetic clusters (heterogeneity *p*<0.05 and *I*^2^>75%). (**b**) Heatmap of metabolite association coefficients (β) across type 2 diabetes clusters. Only the five most significantly associated metabolites for each cluster are shown. ^†^ denotes an association with FDR<5%. The single and double asterisks following the metabolite names indicate that the metabolite was putatively annotated. (**c**) PC analysis of metabolite–pPRS associations

Of the 321 metabolites significantly associated with pPRSs, we identified 232 associated only with a pPRS for a single cluster. For each of the remaining 89 metabolites associated with pPRSs for two or more type 2 diabetes clusters, we assessed the heterogeneity of the association effects across clusters using Cochran’s *Q* test and *I*^2^ statistics. Of the 89 metabolites, 26 (29.2%) exhibited significantly heterogeneous associations across type 2 diabetes genetic clusters (*Q* test *p*<0.05 and *I*^2^>75%; Fig. 3a, b and ESM Table 7), which might explain the lack of significant associations of 20 of these metabolites with oPRS (ESM Table 2).

To systematically characterise the heterogeneity of metabolite associations across the eight clusters, we performed PC analysis on the association coefficients of the 321 metabolites with the eight pPRSs. The first PC primarily separated the lipodystrophy and the metabolic syndrome clusters from the other six clusters while the second PC further resolved the residual glycaemic and the liver and lipid metabolism clusters (Fig. 3c). Given shared biological function, the two beta cell-related clusters expectedly remained close to each other. We compared the pPRS–metabolite associations between each pair of type 2 diabetes clusters and found that the pPRS–metabolite associations only showed modest correlation across clusters (Pearson *r* median 0.17, range –0.14 to 0.64; ESM Fig. 6), reinforcing the heterogeneity of metabolite associations between the eight clusters.

To examine the difference in metabolic pathways impacted by each cluster, we performed enrichment analysis of metabolic pathways in the metabolites associated with pPRS for each cluster. Metabolites associated with the pPRSs for the eight type 2 diabetes genetic clusters showed significant enrichment in 33 metabolic pathways (FDR<5%; ESM Table 5), underscoring heterogeneity of potential metabolic signatures underlying the genetic clusters.

### Associations between genetic risk and incident type 2 diabetes are partly accounted for by metabolites

**Fig. 4:**
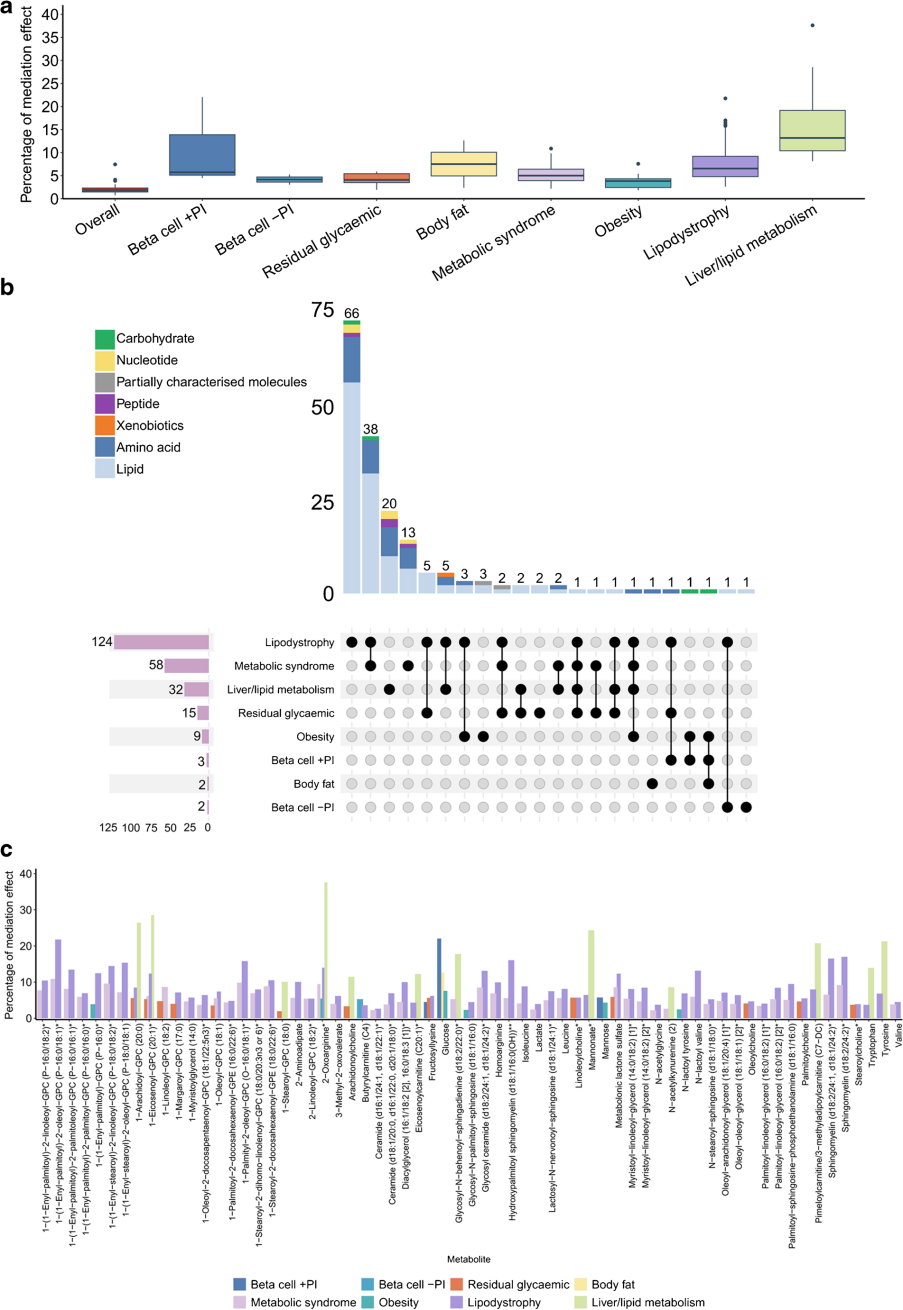
Statistical mediation effects of metabolites for type 2 diabetes genetic risk. (**a**) Comparison of the percentage of metabolite mediation effects for oPRS and the eight pPRSs. In the boxplot, the box represents the interquartile range (25th–75th percentiles), the horizontal line within the box indicates the median, and the whiskers extend to the minimum and maximum values within 1.5 times the interquartile range. (**b**) Upset plot of metabolites showing statistically significant indirect associations. The total number of metabolites with mediation effects for each cluster is shown, along with the number of metabolite mediators shared between clusters and the intersections of clusters. (**c**) Percentage of mediation effect for metabolites that statistically mediated the genetic risk of at least two clusters. The single and double asterisks following the metabolite names indicate that the metabolite was putatively annotated

Of the 95 oPRS-associated metabolites, 59 (62.1%) exhibited statistical patterns consistent with a mediating role in the associations between oPRS and type 2 diabetes incidence (FDR<5%; Fig. 2a). These metabolites statistically accounted for a median of 1.9% of the associations of oPRS with type 2 diabetes incident risk (range of mediation effect 0.7–7.5%; Fig. 4a and ESM Table 8). Of the 321 pPRS-associated metabolites, 171 (53.3%) showed indirect associations that were statistically consistent with a mediating role for the corresponding pPRS (FDR<5%), comprising 245 pPRS–metabolite associations. These indirect associations remained statistically significant after adjusting for HbA_1c_ or fasting plasma glucose (ESM Fig. 7). For the eight type 2 diabetes genetic clusters, 26.2–84.1% of their pPRS-associated metabolites showed indirect associations consistent with a mediating role (FDR<5%; Fig. 4b). These metabolites may partly account for the associations between respective clusters and type 2 diabetes incident risk, with a median of 6.1% of the total effect (percentage range 1.8–37.6%; Fig. 4a, b and ESM Table 8). The magnitude of these indirect associations was significantly greater for pPRS than for oPRS (two-sample *t* test *p*<2.2 × 10^−16^). Joint modelling of metabolites revealed that multiple metabolites together had significantly higher mediation effects than individual metabolites for pPRSs (Wilcoxon rank-sum test *p*=2.5 × 10^−4^; ESM Fig. 8), highlighting a collective contribution of metabolites to the observed associations.

Of the 171 metabolites with statistical patterns suggesting mediation effects for type 2 diabetes pPRSs, 65 (38.0%) showed such patterns for pPRSs of two or more type 2 diabetes genetic clusters, suggesting a potential sharing of metabolic pathways across type 2 diabetes genetic clusters (Fig. 4c). Among them, seven metabolites showed indirect associations consistent with mediating the associations of at least three pPRSs with incident type 2 diabetes. Among the 171 metabolites, 167 (97.7%) remained significant in the statistical mediation analyses after adjusting for type 2 diabetes oPRS (ESM Table 9), reinforcing the extra biological insights gained through type 2 diabetes clustering.

For the 171 metabolites that partly accounted for the associations between pPRSs and type 2 diabetes, our previous metabolite GWAS identified 90 putative causal genes [10]. Of them, 24 (26.7%) were targets of 46 approved or investigational drugs. Notably, one of these genes, *ARG1*, is the target of the investigational drug nor-NOHA for type 2 diabetes (ESM Table 10) [15]. In addition, we identified significant enrichment of gene sets related to 323 drugs in the 90 putative causal genes (FDR<5%; ESM Table 11) using the WebGestalt 2019 as described previously [16, 17]. Notably, gene sets related to the action or metabolism of gemfibrozil and torasemide, two approved treatments for type 2 diabetes [24, 25], achieved the highest enrichment ratios. Of the 90 putative causal genes, five (*CYP1A2*, *CYP2C19*, *CYP2C8*, *CYP2C9* and *SLCO1B1*) were among the gene sets for these two drugs. These results suggest that our findings might help identify potential type 2 diabetes therapeutic targets.

## Discussion

In the present study, we tested associations of 979 named plasma metabolites with type 2 diabetes overall and cluster-partitioned genetic risk from Suzuki et al [3] in 10,015 Finnish men. We identified 95 metabolites significantly associated with type 2 diabetes oPRS. Through partitioning type 2 diabetes genetic risk into eight clusters, we identified a total of 321 metabolite associations, including 242 metabolites not associated with the oPRS. Mediation analysis found that the patterns for more than half of the associated metabolites were consistent with a mediating role between the associations of genetic risk with type 2 diabetes incidence, although their estimated mediation effects were modest, suggesting a complexity of type 2 diabetes metabolic pathways. To our knowledge, this is the first study to comprehensively characterise metabolic signatures for type 2 diabetes genetic clusters, building comprehensive metabolite associations for type 2 diabetes genetic risk and highlighting metabolic heterogeneity across clusters.

Type 2 diabetes clustering is likely essential for precision health in type 2 diabetes. Studies have implicated type 2 diabetes clusters through leveraging participants’ clinical and/or genetic information [3, 7, 26, 27]. The identification of these type 2 diabetes clusters is consistent with heterogeneous disease mechanisms [3, 7, 26, 27]. Type 2 diabetes progression trajectories and complications exhibited differential risk between clusters [3, 28–31], suggesting that the clustering findings can in principle help develop personalised prevention strategies. Recent studies have identified molecules, including proteins and metabolites, associated with type 2 diabetes clusters; however, most studies have focused on type 2 diabetes clustering of clinical and functional characteristics [8, 23, 27, 30, 31].

Here, we profiled plasma levels of 1540 metabolites in more than 10,000 Finnish men of the METSIM study. We systematically evaluated plasma metabolite associations with the genetic risk of the eight clusters recently identified in the largest type 2 diabetes GWAS to date [3]. Of the 321 metabolites associated with type 2 diabetes pPRSs, the majority were specific to a single genetic cluster and they implicated distinct metabolic pathways across clusters. A typical example is the beta cell + PI and beta cell − PI clusters, both of which represented genetic risk relevant to pancreatic beta cell function [3]. Metabolites linked to these two clusters showed significant enrichment in distinct metabolic pathways: the beta cell + PI cluster in fructose, mannose and galactose metabolism; and the beta cell − PI cluster in branched-chain amino acid metabolism. For the 89 metabolites significantly associated with type 2 diabetes genetic risk in two or more clusters, 26 exhibited opposite association direction or significant heterogeneity in their association effects across different clusters, suggesting the complexity of type 2 diabetes biology. For example, of the 26 metabolites, cyclo(leu-pro) can be produced by various species of *Lactobacillus* and exhibits antimicrobial, antioxidant and anticancer effects [32]. Previous studies have found that cyclo(leu-pro) can significantly reduce postprandial plasma glucose levels [33] but no studies have reported direct evidence for its association with type 2 diabetes. In our study, we identified significant associations of plasma cyclo(leu-pro) levels with pPRSs for the body fat and the lipodystrophy clusters, but in opposite directions. This might explain its insignificant association with type 2 diabetes oPRS and suggests its potentially complicated role in type 2 diabetes genetic mechanisms. All these results underscore the potential metabolic heterogeneity across type 2 diabetes genetic clusters.

We leveraged the mediation analysis and the up to 18 years’ follow-up data in METSIM to evaluate the role of metabolites in the associations of genetic risk with type 2 diabetes incidence. We found that more than half of the PRS-associated metabolites showed indirect associations statistically consistent with a mediating role in the association between genetic risk and type 2 diabetes onset, pointing to potentially novel metabolic pathways related to type 2 diabetes genetic susceptibility. We identified significant effects for well-known type 2 diabetes-associated metabolites. For example, leucine and fructosyl-lysine levels have been widely reported to be associated with type 2 diabetes [18, 34–36]. We detected their significant associations with type 2 diabetes genetic risk, which were directionally consistent with previous epidemiological studies [18, 34–36]. Leucine, an essential branched-chain amino acid, regulates insulin secretion from pancreatic beta cells through the mTOR pathway [36]. Fructosyl-lysine, an alpha amino acid, is a biomarker for the assessment of glycaemic management and the risk prediction of diabetes vascular complications [34]. We identified significant indirect effects consistent with a mediating role for both metabolites in the associations of genetic predisposition with incident type 2 diabetes. These results further support the potential of these established type 2 diabetes-related metabolites as intermediate players in the disease mechanism. Our study revealed higher statistical mediation effects of metabolites for pPRSs than for the oPRS. For example, *N*-lactoyl leucine has been implicated in type 2 diabetes pathogenesis [21]. In our study, the indirect association accounted for a substantially greater percentage of incident type 2 diabetes associations with the pPRS for the lipodystrophy cluster (16.0%) than with the oPRS (2.8%), suggesting that *N*-lactoyl leucine may be relevant to the lipodystrophy in type 2 diabetes mechanisms.

Previous studies have identified significant negative associations of plasma *N*-acetylglycine with type 2 diabetes [37] but the role of *N*-acetylglycine in type 2 diabetes genetic mechanisms remains largely unclear. We found that the *N*-acetylglycine level was significantly negatively associated with type 2 diabetes. This association pattern was consistent with a mediating role in the association with type 2 diabetes genetic risk in the metabolic syndrome cluster. The metabolic syndrome cluster includes rs10427109, located near the *INSR* gene, that plays a role in neuronal function and metabolic regulation [38]. *N*-Acetylglycine is a derivative of glycine that is an important inhibitory neurotransmitter [39]. Certain neurotransmitters are present in the islets and are linked to beta cell dysfunction and type 2 diabetes development [40]. On the other hand, insulin receptors are also found in neurons, and insulin resistance can alter neurotransmitter homeostasis, which is associated with neurological complications of type 2 diabetes [41]. Our findings suggest a role of neurotransmission in type 2 diabetes aetiology. Through integrating our mediation analysis findings and previous putative causal gene nominations from metabolite GWAS [10], our study identified a target (i.e. the *ARG1* gene) for type 2 diabetes investigational drugs [15]. These results suggest that the PRS–metabolite associations could help prioritise drug targets, especially when we have improved understanding of the genetic regulation of metabolite levels.

Our study has several limitations. First, we analysed a single cohort of Finnish men. Consequently, the findings may not be directly generalisable to women or to populations of other ancestries. Independent replications in women and other populations are warranted. Second, we identified metabolites partly accounting for the associations of genetic risk with type 2 diabetes incidence, which may help generate metabolic pathway hypothesis. However, the observational nature of our study precludes type 2 diabetes mechanistic exploration and direct causal inference for the metabolite associations. We have not evaluated the assumptions of the mediation analyses and the influence of unmeasured confounding. The potential causal links and the specific metabolic mechanisms require further investigation. Third, we used genetic findings from the largest cross-ancestry type 2 diabetes GWAS meta-analysis for PRS construction [3]. METSIM is an extremely small part of this meta-analysis (<0.5% of the total sample size), which may introduce mild overfitting. Fourth, our study might suffer from statistical power because of the relatively small study sample size.

In summary, our study elucidated the metabolic signatures for type 2 diabetes genetic risk and characterised heterogeneous metabolite associations across type 2 diabetes clusters. The findings have the potential to help develop personalised interventions and tailored-management for type 2 diabetes.

## Supplementary Information

Below is the link to the electronic supplementary material.

Supplementary file1 (PDF 13.9 MB)

Supplementary file2 (XLSX 1225 KB)

## Data Availability

The datasets generated during and/or analysed in the current study are available in dbGaP (accession ID: phs000743.v4.p1 and phs004033.v1.p1).

## Abbreviations

FDR: False discovery rate
GWAS: Genome-wide association study
METSIM: Metabolic Syndrome in Men
oPRS: Overall PRS
PC: Principal component
PI: Proinsulin
pPRS: Genetic cluster-partitioned PRS
PRS: Polygenic risk score
