# Supplementary material for: Plasma metabolite association profiles for type 2 diabetes genetic clusters in Finnish men
Source: Diabetologia. 2026 Mar 18;69(7):1869–81. doi: 10.1007/s00125-026-06710-9 (PMC13236824; doi:10.1007/s00125-026-06710-9)

ESM Figure 1: Comparison of the oPRS-metabolite associations in all METSIM participants (n=10,015; all-individuals), participants without baseline T2D (n=8620), participants with normal glucose tolerance (NGT; n=5744), and participants with baseline T2D (n=1395). “Both Sig” indicates that the oPRS–metabolite associations were significant in both the all-individuals and the subset analyses; “All Individuals Sig” indicates that the associations were only significant in the all-individuals analysis; “Neither Sig” indicates that the associations were insignificant in both the all-individuals and the subset analyses; the remaining label indicates that the associations were significant only in the subset analyses. Concordant indicates “directionally consistent and significantly associated”: the values in the table represent the number of oPRS–metabolite associations that were directionally consistent and statistically significant across analyses (with the number of unique metabolites shown in parentheses).

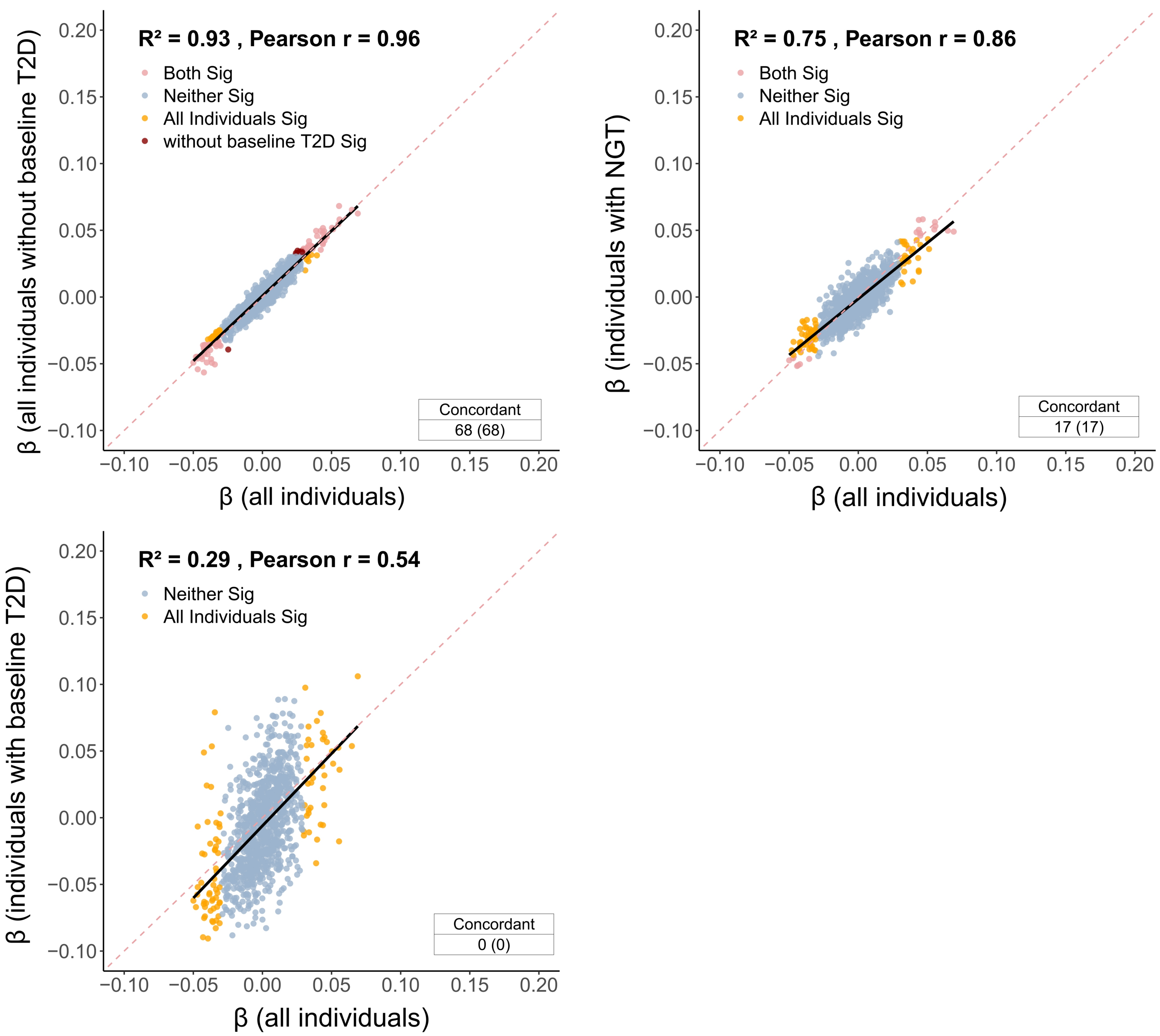

ESM Figure 2: Comparison of pPRS-metabolite associations in all METSIM participants (n=10,015; all-individuals), participants without baseline T2D (n=8620), participants with normal glucose tolerance (NGT; n=5744), and participants with baseline T2D (n=1395). “Both Sig” indicates that the pPRS–metabolite associations were significant in both the all-individuals and the subset analyses; “All Individuals Sig” indicates that the associations were only significant in the all-individuals analysis; “Neither Sig” indicates that the associations were insignificant in both the all-individuals and the subset analyses; the remaining label indicates that the associations were significant only in the subset analyses. Concordant indicates “directionally consistent and significantly associated”: the values in the table represent the number of pPRS–metabolite associations that were directionally consistent and statistically significant across analyses (with the number of unique metabolites shown in parentheses).

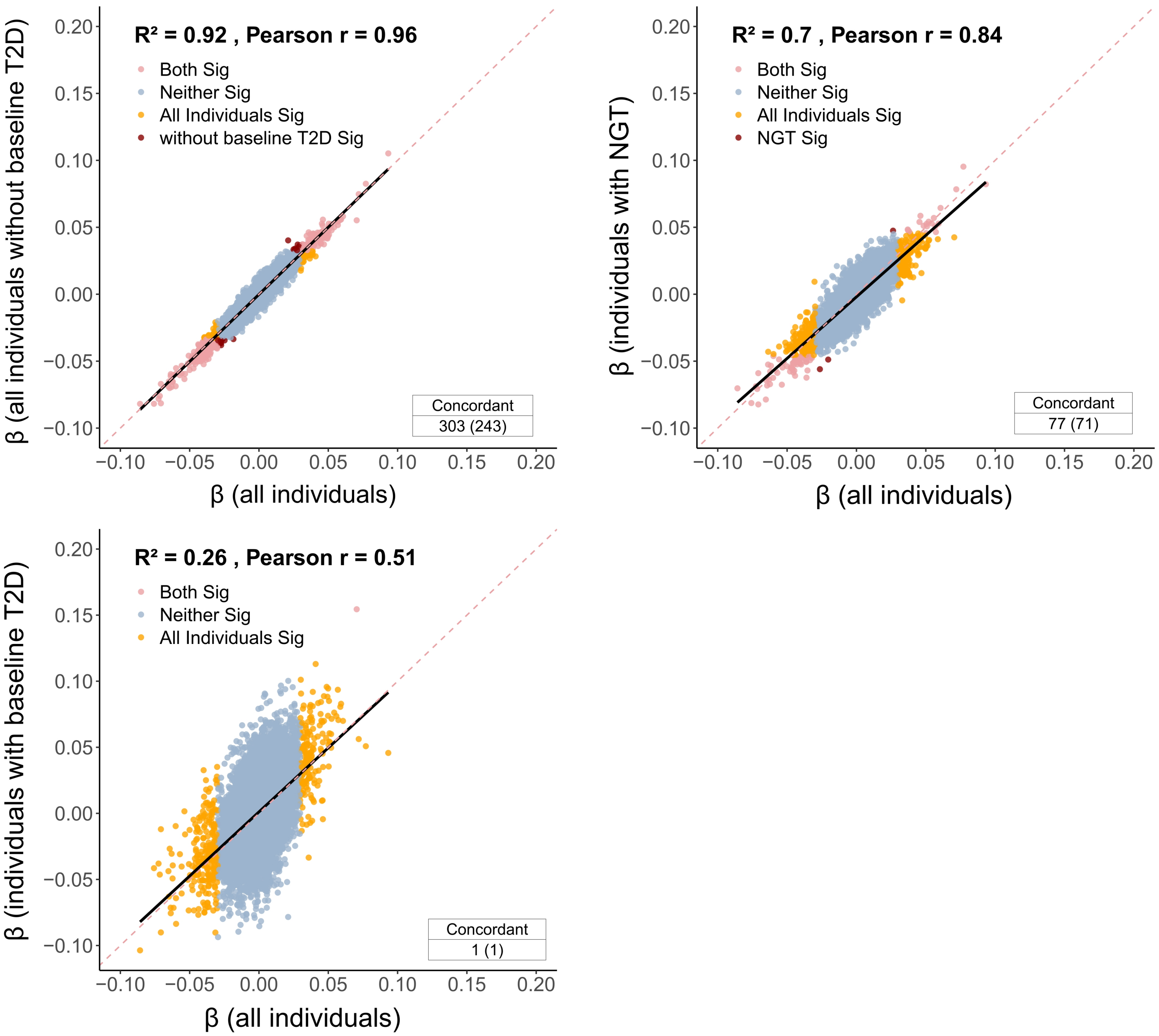

ESM Figure 3: Correlation between the number of significantly-associated metabolites with a pPRS and the number of genetic variants within the respective cluster. The dashed line in red represents the diagonal line. The solid line in gray denotes the regression line. SNV: single nucleotide variant.

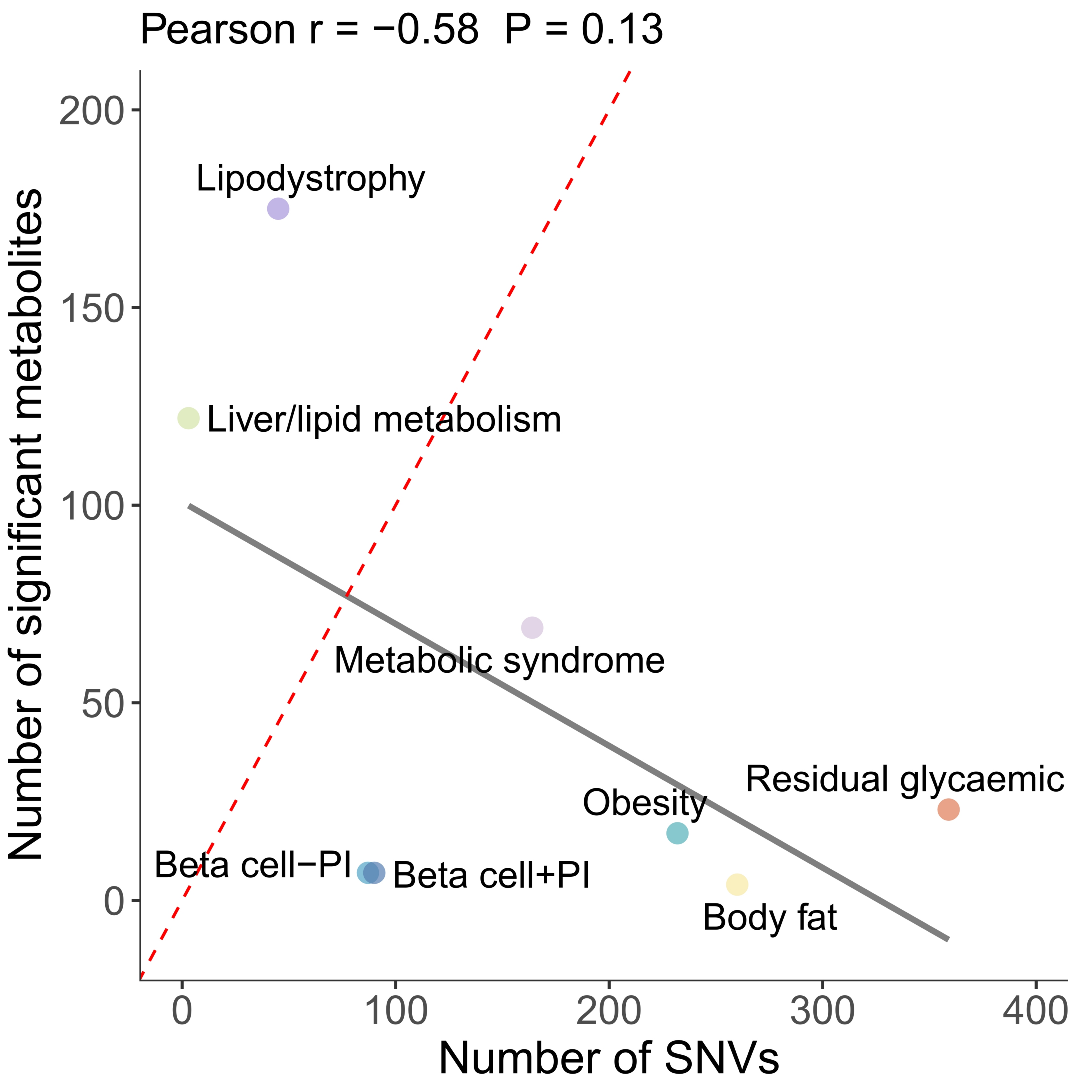

ESM Figure 4: Correlations between the total acyl chain carbon number or double bond content of lipids and their association coefficients with pPRS. The *x*-axis represents the total acyl chain carbon number or the double bond content of a lipid, and the *y*-axis represents the association coefficient of the lipid with pPRS. The red line denotes the regression line with the 95% confidence interval as the gray shaded area. The *p*-values from Spearman's rank correlation test is on the top. The metabolic pathway of the lipids and the cluster name are in red below the plot.

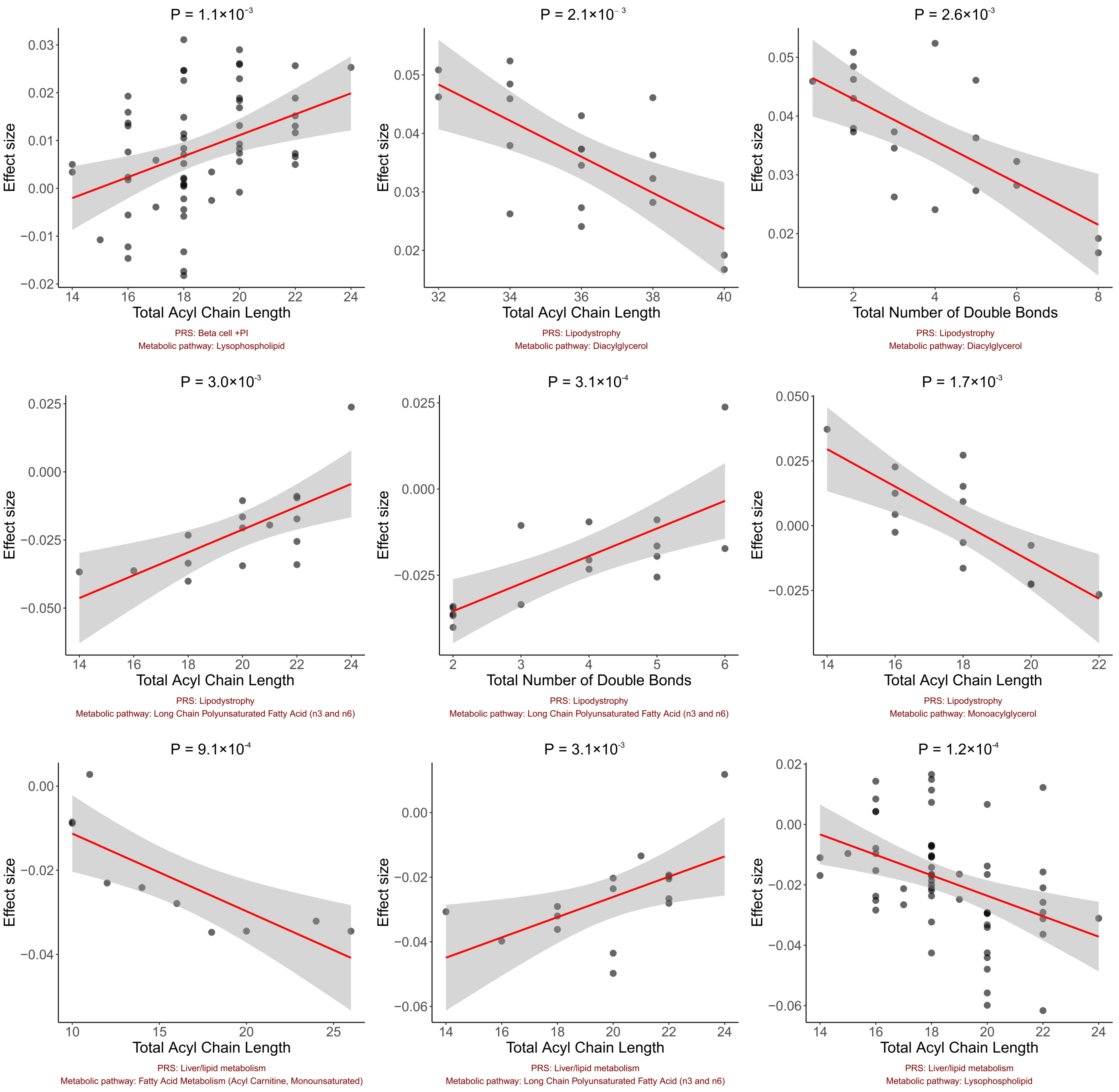

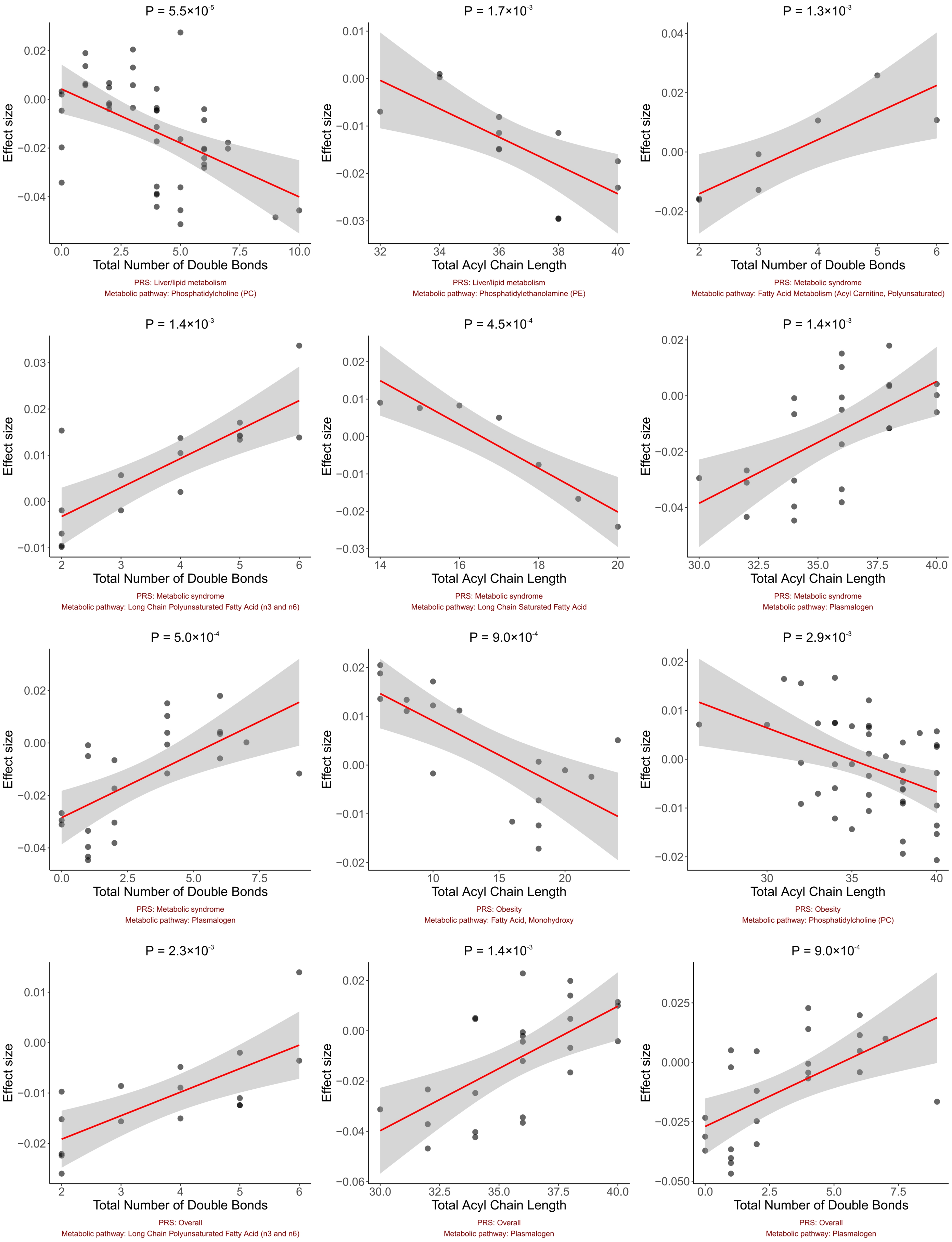
P = 5.5×10-5
Effect size
Total Number of Double Bonds
PRS: Liver/lipid metabolism
Metabolic pathway: Phosphatidylcholine (PC)
P = 1.7×10-3
Effect size
Total Acyl Chain Length
PRS: Liver/lipid metabolism
Metabolic pathway: Phosphatidylethanolamine (PE)
P = 1.3×10-3
Effect size
Total Number of Double Bonds
PRS: Metabolic syndrome
Metabolic pathway: Fatty Acid Metabolism (Acyl Carnitine, Polyunsaturated)
P = 1.4×10-3
Effect size
Total Number of Double Bonds
PRS: Metabolic syndrome
Metabolic pathway: Long Chain Polyunsaturated Fatty Acid (n3 and n6)
P = 4.5×10-4
Effect size
Total Acyl Chain Length
PRS: Metabolic syndrome
Metabolic pathway: Long Chain Saturated Fatty Acid
P = 1.4×10-3
Effect size
Total Acyl Chain Length
PRS: Metabolic syndrome
Metabolic pathway: Plasmalogen
P = 5.0×10-4
Effect size
Total Number of Double Bonds
PRS: Metabolic syndrome
Metabolic pathway: Plasmalogen
P = 9.0×10-4
Effect size
Total Acyl Chain Length
PRS: Obesity
Metabolic pathway: Fatty Acid, Monohydroxy
P = 2.9×10-3
Effect size
Total Acyl Chain Length
PRS: Obesity
Metabolic pathway: Phosphatidylcholine (PC)
P = 2.3×10-3
Effect size
Total Number of Double Bonds
PRS: Overall
Metabolic pathway: Long Chain Polyunsaturated Fatty Acid (n3 and n6)
P = 1.4×10-3
Effect size
Total Acyl Chain Length
PRS: Overall
Metabolic pathway: Plasmalogen
P = 9.0×10-4
Effect size
Total Number of Double Bonds
PRS: Overall
Metabolic pathway: Plasmalogen

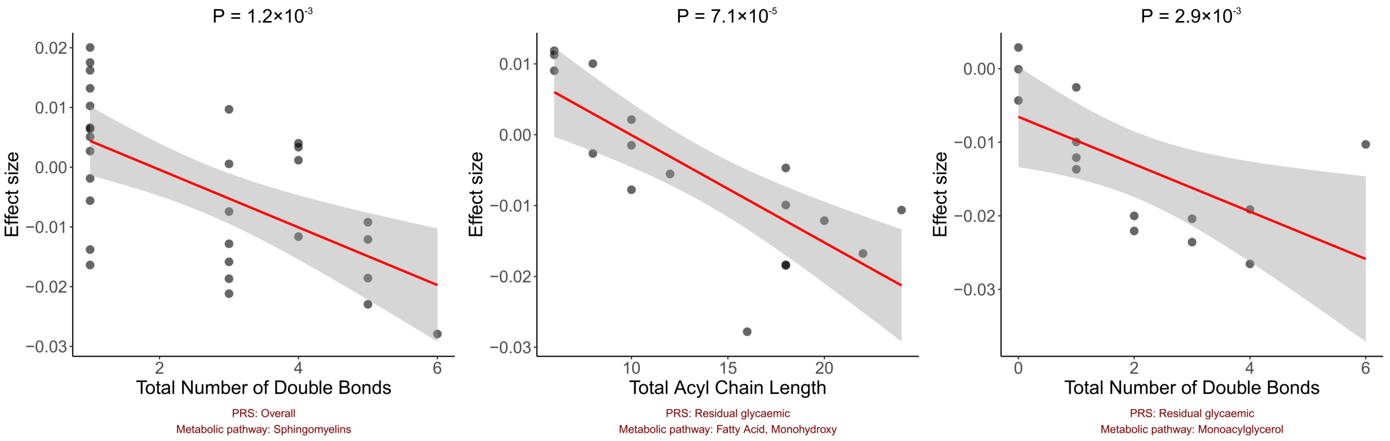
P = 1.2×10⁻³
Effect size
Total Number of Double Bonds
PRS: Overall
Metabolic pathway: Sphingomyelins
P = 7.1×10⁻⁵
Effect size
Total Acyl Chain Length
PRS: Residual glycaemic
Metabolic pathway: Fatty Acid, Monohydroxy
P = 2.9×10⁻³
Effect size
Total Number of Double Bonds
PRS: Residual glycaemic
Metabolic pathway: Monoacylglycerol

ESM Figure 5: Comparison of the T2D pPRS-metabolite associations between with and without oPRS adjustment. “Both Sig” indicates significant pPRS-metabolite associations in both models with and without oPRS adjustment; “Adj. Sig” indicates associations only significant in the model with adjustment while “Unadj. Sig” indicates associations only significant in the model without adjustment; “Neither Sig” indicates associations that were neither significant in the model with oPRS adjustment nor in the model without oPRS adjustment.

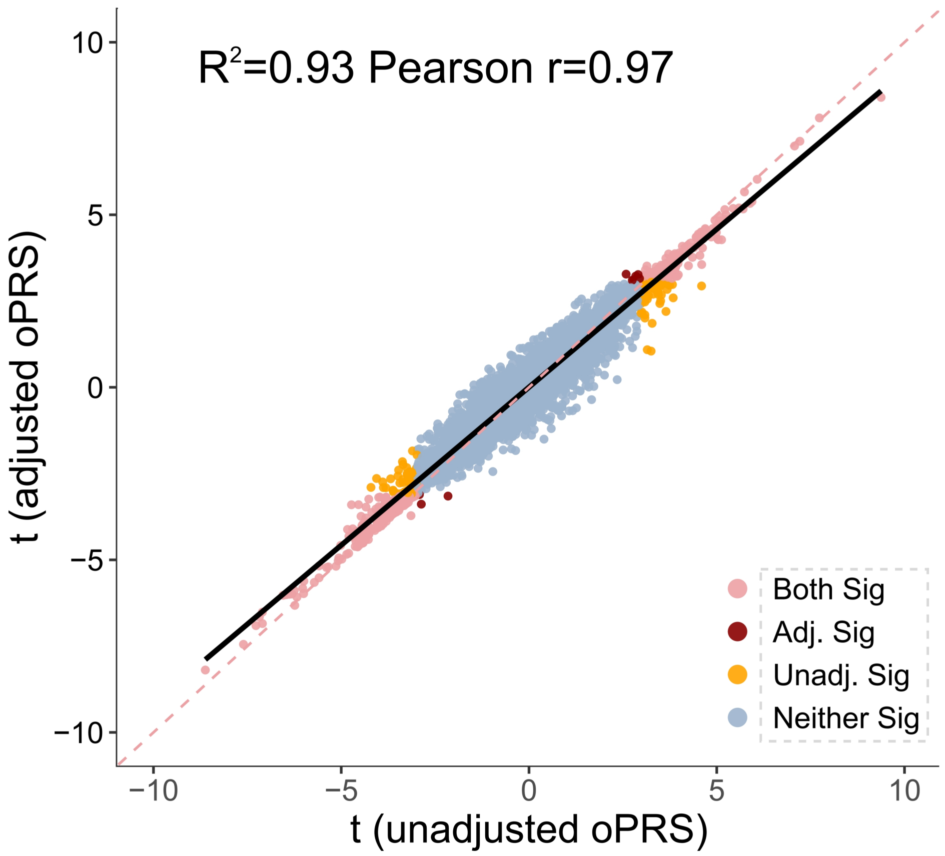

ESM Figure 6: Correlations of associations with all the 979 metabolites (t-values) between each two clusters. The lower triangle shows the scatter plots of the metabolite association t-values between each pair of the eight clusters. The *x*- and the *y*-axis denote the metabolite association t-values with cluster. The solid line in black represents the regression line. The upper triangle shows the Pearson correlation coefficients and their test *p*-values. Stars indicate significant Pearson correlations of metabolites between the two clusters, with one star (*) representing *P*<0.05, two stars (**) representing *P*<0.01, and three stars (***) representing *P*<0.001. The diagonal panels show the density distributions of metabolite association t-values with each of the eight clusters.

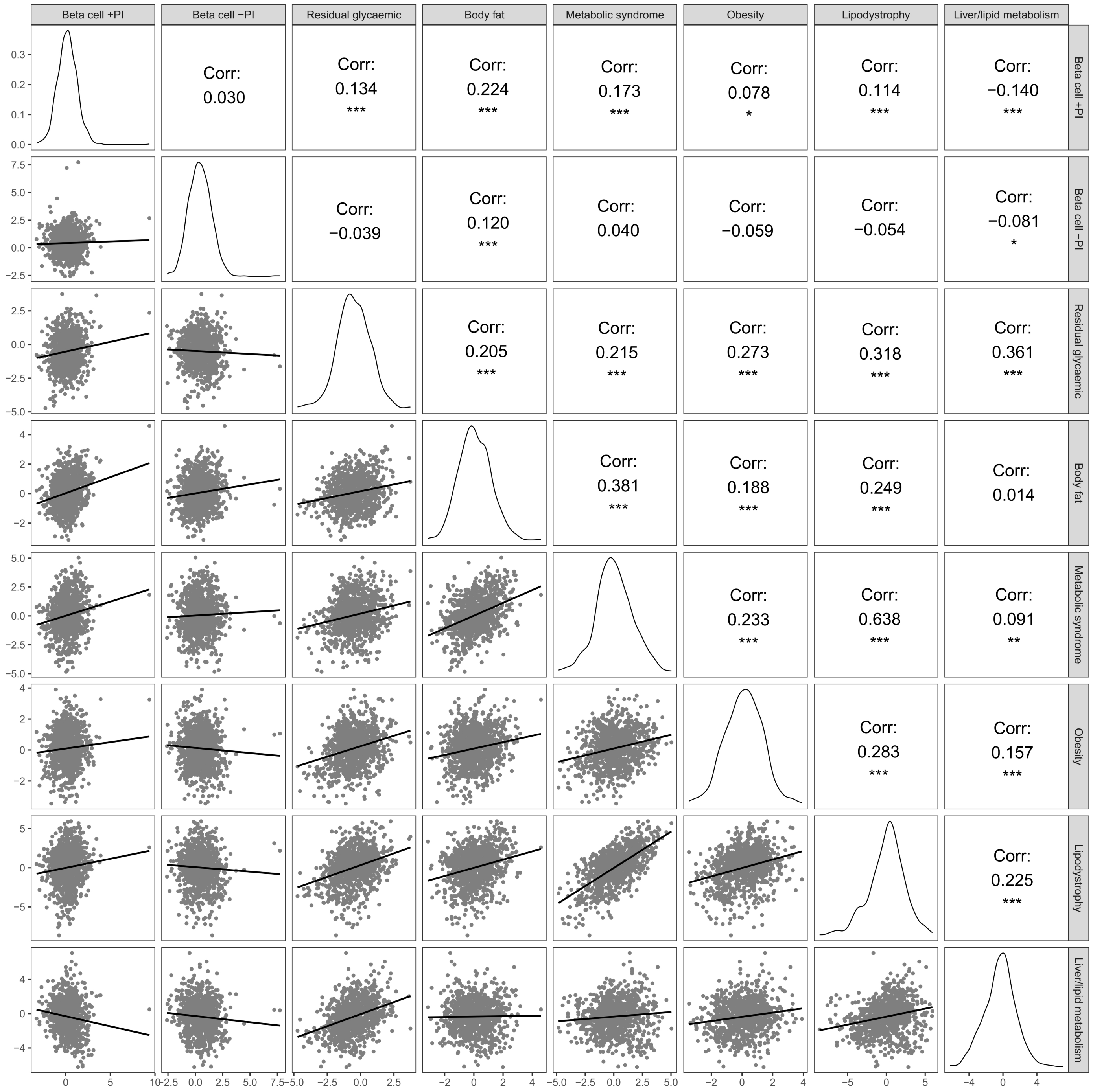

ESM Figure 7: Comparison of statistical mediation effects with and without adjustment for glycated haemoglobin (HbA_{1c}) or fasting plasma glucose (FPG) levels. "Both Sig" indicates mediation effects achieving statistical significance in both the models with and without adjustment while "unadjusted HbA1c/FPG Sig" refers to those significant only without adjustment.

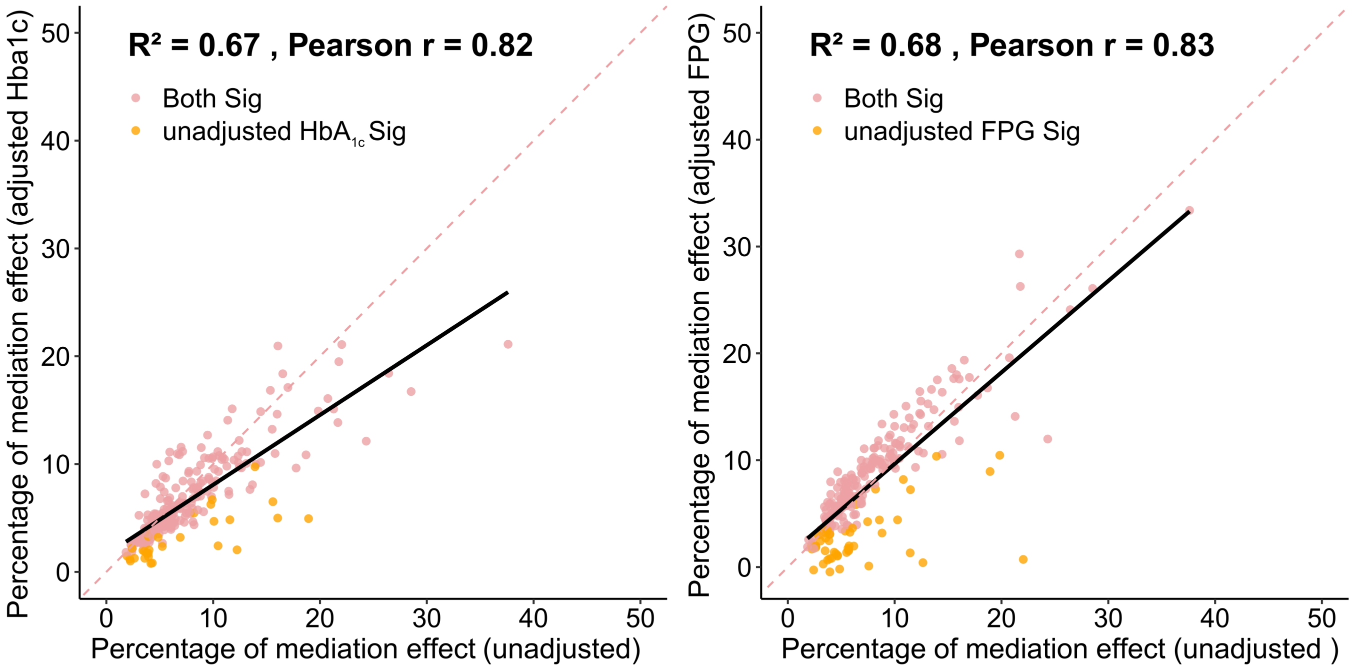

ESM Figure 8: Comparison of statistical mediation effects between multi-metabolite scores and individual metabolites for each pPRS. The boxplots show the mediation percentages of the associations statistically accounted for by individual metabolites for each pPRS, and the triangles represent the mediation percentages of the respective multi-metabolite score for the pPRS-T2D association.

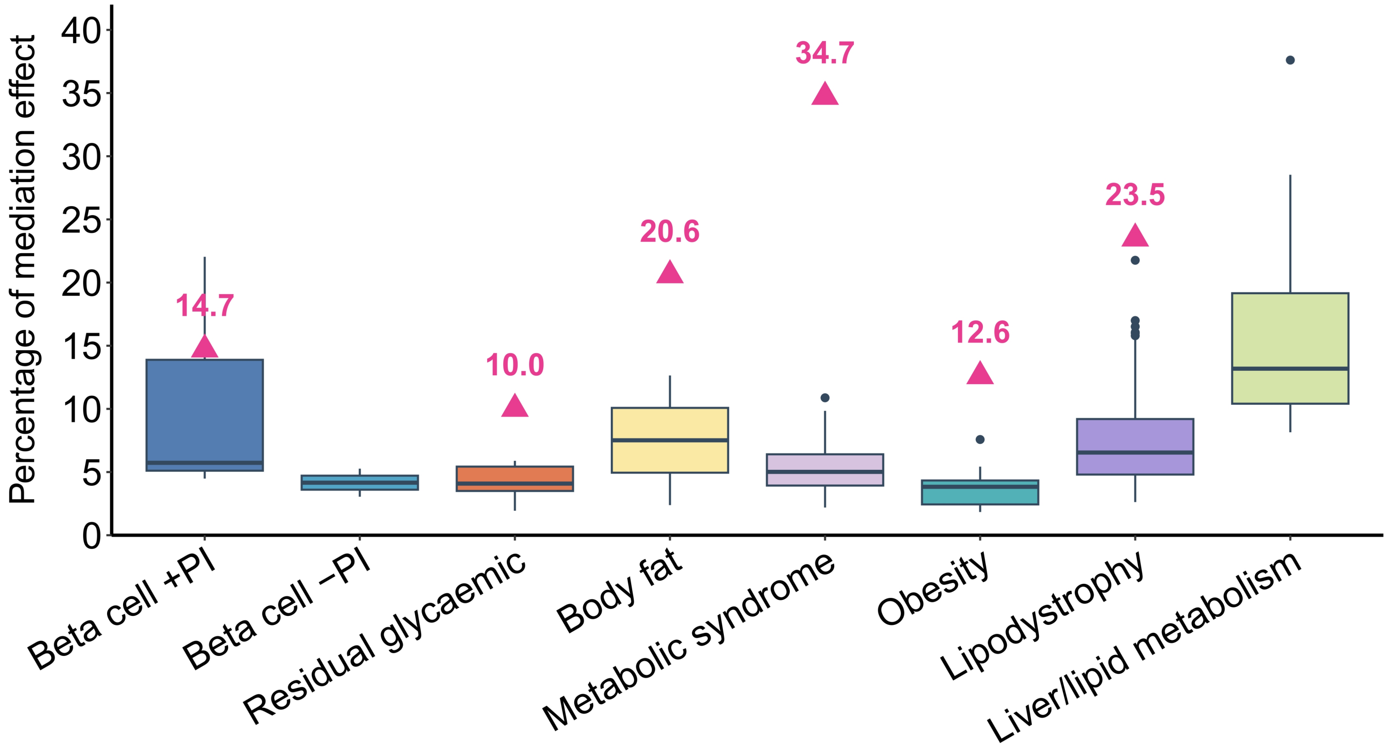

Supplement: Supplementary file 1 — Supplementary file1 (PDF 13.9 MB) [file 125_2026_6710_MOESM1_ESM.pdf]
